# Exposure to soiled bedding reduces abnormal repetitive behaviors in mice

**DOI:** 10.3389/fnbeh.2022.1062864

**Published:** 2022-11-22

**Authors:** Karin Müller, Theresia Lengheimer, Julia B. Kral-Pointner, Johann Wojta, Lusine Yeghiazaryan, Christoph Krall, Rupert Palme, Sonia Kleindorfer, Roberto Plasenzotti, Daniela D. Pollak, Katharina E. Tillmann

**Affiliations:** ^1^Department of Neurophysiology and Neuropharmacology, Center for Physiology and Pharmacology, Medical University of Vienna, Vienna, Austria; ^2^Core Facility Laboratory Animal Breeding and Husbandry, Medical University of Vienna, Vienna, Austria; ^3^Department of Internal Medicine II (Cardiology), Medical University of Vienna, Vienna, Austria; ^4^Center for Medical Statistics, Informatics and Intelligent Systems, Medical University of Vienna, Vienna, Austria; ^5^Unit of Physiology, Pathophysiology and Experimental Endocrinology, University of Veterinary Medicine Vienna, Vienna, Austria; ^6^Department of Behavioral and Cognitive Biology, University of Vienna, Vienna, Austria

**Keywords:** abnormal repetitive behaviors, barbering, bar mouthing, soiled bedding, sentinel mice

## Abstract

Hygiene management protocols in laboratory mouse husbandries worldwide most commonly employ soiled bedding-exposed sentinel mice to monitor the occurrence of infections in mouse colonies. Using this approach, sentinel mice repeatedly receive a mixture of used bedding, supplied by a variety of cages of a defined hygienic unit for a period of several months. Hereby, microorganisms shed in the used bedding can infect the sentinel animals and can be detected in subsequent health monitoring procedures. However, murine excrements carry more than only microorganisms. Mouse feces and urine also contain a multitude of olfactory molecules, which the animals use to code information about social status and context. However, if and how the persistent and repeated experience with these odor cues affects the behavior of sentinel mice, has not yet been explored. To address this question, we conducted a longitudinal study for neurochemical output parameters related to an organism’s responsiveness to challenging conditions, and for the exploratory assessment of a panel of home cage behaviors in soiled bedding and control female C57BL/6J mice. We found that the number of mice showing abnormal repetitive behaviors, including barbering and bar mouthing, was lower in the soiled bedding group. While neutrophil/lymphocyte ratios and fecal corticosterone metabolites did not differ between groups, the within-group variance of the neutrophil/lymphocyte ratio was reduced in the soiled bedding group. These results show that the occurrence of abnormal repetitive behaviors is lower in sentinel than in control mice and suggest a beneficial effect of soiled bedding on the welfare of laboratory mice and on outcome variability.

## Introduction

The purposeful exposure of naïve mice to soiled bedding of various cages of a defined hygienic unit is a routinely employed practice to survey and control the health/infectious status of breeding and experimental animals within laboratory mouse facilities. This approach is based upon the consideration that the repeated contact with used bedding and therein contained feces, results in unavoidable contact of so called “sentinel” mice with potential microbial contamination of the soiled bedding, leading to a serologically detectable infection (Maehler et al., 2014).

While defined recommendations and guidelines specifying protocols and conditions for the sentinel hygiene monitoring program exist and are internationally well accepted (de Bruin et al., 2016), little attention has so far been paid to the effects of soiled bedding on the exposed animals, beyond the infection with bacterial or viral agents. Indeed, urine and feces contain a multitude of odor molecules, which constitute the essence of olfactory communication pathways between rodents. These odor molecules are present in the form of pheromones and signature mixtures. Pheromones are molecules that have evolved into signals that elicit a specific response in the receiver of the message to serve territorial marking, sexual signaling and health status conveyance (Wyatt, 2009). Signature mixtures consist of, albeit not exclusively, pheromones, to compose a highly specific mixture that identifies an individual organism (Wyatt, 2009). Both pheromones and signature molecules are not only present in feces and urine, but also secreted through other scent glands, all of which release into the bedding. As such soiled bedding carries important information about age, sexual and health status, degree of kinship and identity of the animals (Latham and Mason, 2004), which is pivotal for the social organization of groups of individuals within a colony. Provision with dirty bedding has been previously associated with an increase in aggression in male mice (Van Loo et al., 2001). However, in male mice perception of pheromonal cues lead to activation of VN2 receptors for sex recognition and aggression as default response (Dulac and Torello, 2003), while in female mice aggression is elicited only under specific experimental conditions (Newman et al., 2019). First insights into the behavioral and physiological effects of acute and chronic soiled bedding exposure in mice have been recently obtained (Merley et al., 2022). This report describes that providing female mice with a mixture of male and female dirty bedding for a period of 4 weeks only led to a difference in body weight between soiled bedding and control mice. However, locomotor activities and corticosterone responses were not determined in this study. Nevertheless, the consequences of the long-term unavoidable exposure to scent marks of foreign animals, for a time period corresponding to the duration of 12 week, suggested for sentinel mice by international guidelines (Maehler et al., 2014), remain elusive. Here we used a study design adhering to the official recommendations for sentinel mouse programs to characterize the consequences of soiled bedding on behavioral, physiological, and physical parameters and monitor neurochemical proxy indicators of the endogenous challenge response system.

## Materials and methods

### Animals

A total of 120 female C57BL/6J mice (Charles River, Sulzfeld, Germany) were used for the present study (*ARRIVE guideline item 8)*. Mice arrived at the facility at 8 weeks of age and were randomly assigned to the experimental group (soiled bedding) or the control group (fresh bedding) (*ARRIVE guideline item 1a)*, using the software “Randomizer” of the Institute for Medical Informatics, Statistics and Documentation of the Medical University of Graz, Austria (*ARRIVE guideline item 4a).* To minimize potential confounders like the order of treatment, cage allocation in rack was determined before the allocation to the study groups (*ARRIVE guideline item 4 b).* 60 animals of each group were housed in specially adapted Makrolon Type III cages (three animals per cage) for feces sampling (Supplementary Figure 1) the other 60 animals of each group were kept in standard Makrolon Type III cages (three animals per cage) (*ARRIVE guideline item 2a*, see also Figure legends). Standard cages were used in order to more readily transfer potential pathogens as recommended for sentinel mice. The weekly cage changes were performed 8–10 h before the start of the dark phase. The housing room was kept at a constant temperature of 22 ± 2°C, humidity 55 ± 10%, and 12 h: 12 h day/night rhythm (lights on at 7 a.m.). All animals received a standard maintenance diet (Mäuse-Haltung, autoclavable; LASQCdiet^®^ Rod16, Auto, 10 mm, Zero, Cert * LasVENDI, Germany) and untreated tap water *ad libitum*. Every cage was equipped with nesting material (sizzlenest, Datesand, Manchester, UK), 12 cellulose swabs (Lohmann & Rauscher International, Rengsdorf, Germany) and aspen wood bedding (ABEDD-LAB & VET Service, Vienna, Austria). Nesting material was entirely renewed at every weekly cage change. The animals were tested for their SPF-status according to FELASA guidelines (Maehler et al., 2014) at the start and at the end of the study and tested negative for all pathogens.

The project was approved by the ethics and animal welfare committee of the Medical University of Vienna in accordance with the national legislation (license number BMBWF-66.009/0257- V/3b/2018). The study was performed according to the ARRIVE guidelines.

### Soiled bedding

Soiled bedding was prepared by collecting and thoroughly mixing bedding (without nesting material) from all 40 cages of the experimental room (only female animals) at the weekly cage change. In the soiled bedding group half of the clean bedding was removed (without the nesting material) and replaced with the mixture of soiled bedding (*ARRIVE guideline item 9*).

### Home cage behavior

**FIGURE 1:**
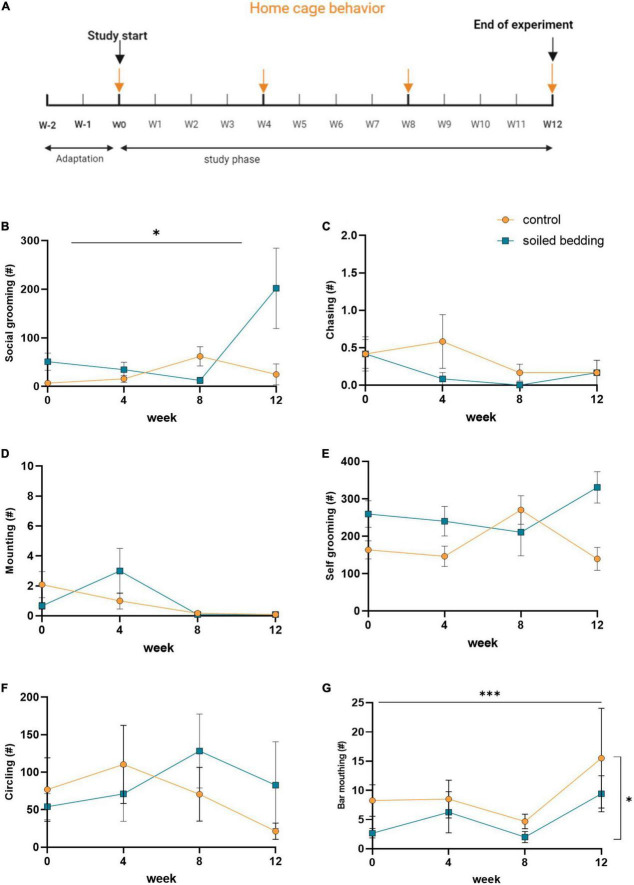
Home cage behavioral observation in soiled bedding and control mice. **(A)** Timeline for the video-based home cage evaluation. Arrows indicate days at which home cage behavior was monitored. **(B–D)** Evaluation of social behaviors: **(B)** Frequency of social grooming, **(C)** chasing behavior, and **(D)** mounting behavior in control and soiled bedding groups (*n* = 12/group). **(E–G)** Evaluation of non-social behaviors: **(E)** Frequency of self-grooming, **(F)** circling, and **(G)** bar mouthing behavior in control and soiled bedding groups (*n* = 12/group). All data are displayed as mean ± SEM. Only main effects of treatment and time × treatment effects are indicated: *p < 0.05, ^***^p < 0.001.

Video observation in the home cage was performed for 24 h at four times during the study period, randomly selecting four cages per group (Figure 1A). The cages were filmed with HiLook NVR-108MH-D/W eight channel network video recorders and eight matching power over ethernet IP-cameras. Behavioral parameters scored included social (social grooming, chasing, and mounting) and non-social behaviors (self-grooming, circling, and bar mouthing) (Garner et al., 2021). Each mouse was evaluated separately (*n* = 12/group) for a period of 30 min between 9 and 11 p.m. using the Solomon coding software (Péter, 2021) (Solomon Coder © 2019 by András Péter, Version: beta 19.08.02) by a trained observer blinded to the experimental conditions of the animals (*ARRIVE guideline item 5*).

### Physical markers

#### Weekly cage change

**FIGURE 2:**
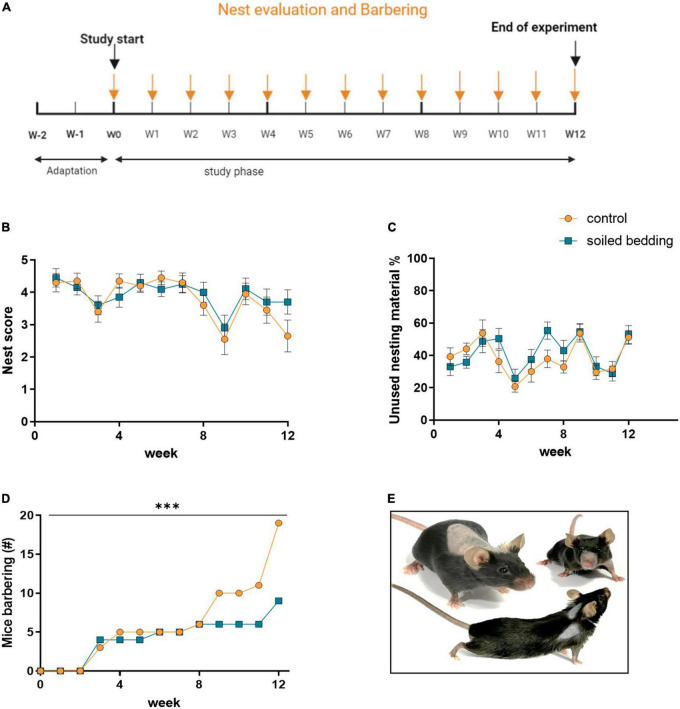
Evaluation of physical markers in soiled bedding and control mice. **(A)** Timeline for the evaluation of physical markers throughout the study. **(B)** Evaluation of the nest quality (nest score) in soiled bedding and control cages (*n* = 20 cages/group). **(C)** Amount of unused nesting material in soiled bedding and control cages (*n* = 20 cages/group). **(D)** Number of mice exhibiting barbering marks in control and soiled bedding groups over the course of the study (*n* = 60/group). **(E)** Exemplary images illustrating frequently observed barbering marks. All data are displayed as mean ± SEM. Only main effects of treatment and time × treatment effects are indicated: ^***^p < 0.001. Arrows indicate days at which the nest and barbering marks were evaluated.

24 h after the cage the nests of each cage were photographed (Figure 2A). The images were scored for the quality of the nest (Hess et al., 2008) by a blinded observer. The score applied is suggested as indicator for mouse health and welfare (Gaskill et al., 2013) and can be shortly explained as follows: “0”: no used nesting material, “1”: nesting material was moved but not gathered to a nest site, “2”: nesting material was moved to a nest site but appears flat, “3”: cup-shaped nest, “4”: nest shaped as incomplete dome, “5” nested shaped as complete dome. Additionally, unused cellulose swabs were counted.

#### Physical appearance of the mice

During the weekly cage change the physical appearance of all animals was investigated (Figure 2A). Deviations of the normal appearance were scored for the following parameters:

• *Coat state*: unkemptness or signs of barbering
• *Tail and paws*: injury or swelling
• *Eyes, nose, and mouth*: discharge or signs of infections

### Physiological evaluations

#### Body weight

The weight of all mice was assessed every four weeks during the cage cleaning process.

#### Fecal corticosterone metabolites

Fecal corticosterone metabolites (FCMs) were measured four times during the study period using samples collected from the custom-made sampling cages (Supplementary Figure 1; Touma et al., 2004; Frynta et al., 2009). Prior to the start of the study mice were trained to enter the sampling boxes.

#### Neutrophil: Lymphocyte ratio

On study day 85, all mice were anesthetized with 0.5 mg/kg Medetomidine, 5.0 mg/kg Midazolam i.p. and 0.05 mg/kg Fentanyl i.p., injection volume 0.1 ml/10 g of body weight, and blood was taken *via* cardiac puncture and anticoagulated with dipotassium ethylenediaminetetraacetic acid (K2EDTA). Immediately after, animals were euthanized *via* cervical dislocation. To calculate the N/L ratio whole blood was stained with respective antibodies for 15 min and after 15 min fixation with 1% formaldehyde, red blood cells were lysed with BD FACS Lysing Solution (BD Biosciences, San Jose, CA, USA). Afterward, the cells were centrifuged for 5 min at 500 × g, and the supernatant was discarded. The pellet was re-suspended in 1% formaldehyde, acquired by flow cytometry (Attune^®^ NxT Acoustic Focusing Cytometer, Thermo Fisher Scientific, Austria, Vienna) and analyzed by Attune™ NxT Software (Thermo Fisher Scientific, Austria, Vienna). The following anti-mouse antibodies were used: α-CD3e-AF488, α-CD11b-PerCP, α-B220-BV421, α-Ly6G-APC, α-CD45-BV650, (all Biolegend, San Diego, CA, USA). Neutrophils were defined as CD45^+^, CD11b^+^ Ly6G^+^ cells, lymphocytes were identified by CD45^+^, CD11b^–^ and verified by positivity for either B220 or CD3e.

### Statistics

Sample size calculation was conducted using the N/L ratio as primary endpoint, with the N/L ratio as dependent variable and treatment (control/soiled bedding) as independent variable (*ARRIVE guideline item 2b, 6a and b*). Single animals were considered as experimental unit (*ARRIVE guideline item 1b*). No animals were excluded from statistical analysis (*ARRIVE guideline item 3a and b, for 3c*, see Figure legends). The difference in variance was analyzed based upon an F-test. For FCM mixed models with random effect mouse nested in cage and fixed effects litter-group and time with interaction were calculated to determine whether the values in the control group and in the treatment group differed over time. For behavioral outcomes random coefficient Poisson models with random effect mouse nested in cage and fixed effects litter-group and time with interaction were calculated. All calculations were conducted using R (© The R Foundation. version 4.0.3; Function lmer() from the package “lme4”). Analyses of behavioral data, physical marks and body weight was conducted in an explorative manner and naïve *p*-values are considered. Hypothesis-based testing was conducted for FCM and N/L ratio (*ARRIVE guideline item 7*).

## Results

The objective of the present study was to evaluate the long-term consequences of soiled bedding exposure, mimicking the conditions of sentinel animals. The experimental design of the study was therefore planned in accordance with the FELASA recommendations for sentinel hygiene monitoring programs (Maehler et al., 2014). Aiming to characterize the consequences of 12 weeks continuous soiled bedding exposure, selected behavioral, physical, and physiological parameters with relevance to animal well-being and the challenge response system were evaluated. We focused on reporting main effects of treatments and/or time × exposure condition interactions in the results and figures. A full description of all statistical results is provided in Supplementary Table 1 (*ARRIVE guideline item 10*).

### Home cage behavior

The home cage behavior of all animals was evaluated at four times during the 12-weeks observational period (Figure 1A). Parameters of social (grooming, chasing, mounting) and non-social behavior (self-grooming, circling, and bar-mouthing) revealed an increase in social grooming selectively at the end of the study period in the soiled bedding group (time × treatment effect; *p* = 0.036; Figure 1B). No other differences between groups were detected for chasing and mounting behavior (Figures 1C,D). For non-social behaviors there was no effect of soiled bedding on self-grooming or circling behavior (Figures 1E,F), but a reduction of bar-mouthing behavior in soiled bedding compared to control mice (time × treatment effect; *p* = 0.027; Figure 1G). These observations provide evidence that the long-term exposure to soiled bedding is associated with an increase in non-agonistic social behavior and a concomitant reduction of bar-mouthing, a stereotypic behavior indicative for the motivation of the animal to escape (Nevison et al., 1999; Würbel et al., 2010).

### Barbering marks

The examination of physical marks included the weekly scoring of the nest (and unused nesting material) and the determination of barbering marks (Figure 2A). The nest score and amount of nesting material used is shown in Figures 2B,C. Differences between groups seem to be much smaller than differences between time points. However, the conspicuous correlation between the two groups suggests the presence of unknown confounders, therefore we abstain from further statistical analysis. Importantly, a lower number of animals displayed was observed, barbering marks (Figure 2D) in the soiled bedding exposed group, specifically at the later time points when overall the occurrence of barbering marks in animals was increased (time × treatment effect; *p* < 0.0001; Figure 2E).

In neither group deviations from the physiological state of the tail and paws or eyes, nose, and mouth were noted.

#### Neurochemical readouts indicating responsiveness to challenging conditions

**FIGURE 3:**
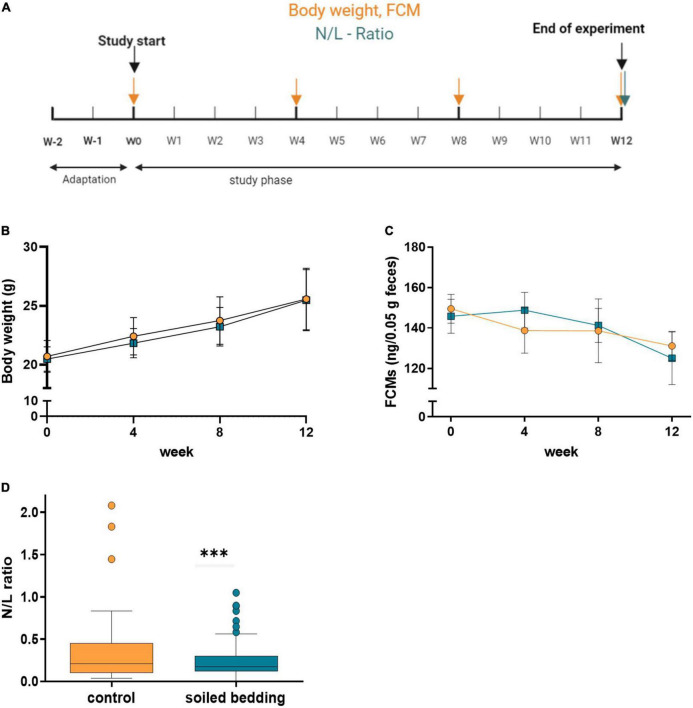
Evaluation of physiological parameters in soiled bedding and control mice. **(A)** Experimental timeline for the evaluation of physiological parameters in soiled bedding and control mice. **(B)** Body weight (g) in animals of the control and soiled bedding groups over the course of the study (*n* = 60/group). **(C)** FCM (ng/0.05 g feces) in mice of the control and soiled bedding groups (*n* = 9–25/group). **(D)** N/L–Ratio: Variance of N/L ratio in the soiled bedding and control groups (*n* = 48–51/group). All data are displayed as mean ± SEM. Only main effects of treatment and time × treatment effects are indicated: ^***^p < 0.001. Yellow arrows indicate days at which body weight and FCM were measured; the blue arrow indicates the day of N/L ratio assessment.

Selected physiological markers (body weight, FCM) for the assessment of the animals’ well-being and their challenge response system were determined every four weeks L/N ratio was measured at the end of the observation period (Figure 3A). We found no difference in body weight or FCM between groups, but a decrease of FCM levels over time in both groups (Figures 3B,C).

Interestingly, while the average N/L ratio was comparable in soiled bedding and control animals, we found that the variance within the soiled bedding groups was significantly lower than in the control group (*p* = 0.005; Figure 3D).

These data suggest that while soiled bedding may not modulate the overall challenge response in a cohort of mice. It may serve to reduce individual variability.

## Discussion

It has previously been shown in male mice that switching animals to the used cage of another animal or transferring different parts of used bedding (i.e., urine and feces contaminated litter or nesting material) constitutes a source of psychosocial stress and influences aggressive behavior (Van Loo et al., 2000, Lee et al., 2004a,b). It has to be pointed out that this effect has to be considered as sex-specific as in male mice the exposure to pheromonal signals induces aggression as default response (Dulac and Torello, 2003) while this effect occurs in females only in particular circumstance (Newman et al., 2019).

However, these reports suggest the possibility that the exposure to soiled bedding could also impact the physiological responsiveness to challenge situations and affect behavior of sentinel mice, a question that had not been addressed so far. Along these lines, a first study on the acute and chronic consequences of dirty bedding provision was published only recently (Merley et al., 2022). Nevertheless, the effects of long-term subjection to soiled bedding, in a time frame comparable to the protocol of sentinel animals, still remained elusive.

We here sought to investigate if and how long-term exposure to soiled bedding, in an experimental design naturalistically reproducing the conditions of sentinel mice as designated by internationally accepted standards of hygiene monitoring, affected the well-being and challenge responsiveness of the animals. To this end we selected behavioral, physical, and physiological read-outs and found that soiled bedding provision increased non-agonistic social behaviors, while reducing some behavioral stereotypies and decreasing the between-animal variability in a hematological parameter associated chronic stress (Davis et al., 2008; Swan and Hickman, 2014; Hickman, 2017).

The observation that the long-term contact with soiled bedding increased social grooming but decreased bar-mouthing behavior and barbering is indicative of a potential positive effect on animal well-being. It has to be pointed out that animals exhibiting signs of barbering are used as a proxy to evaluate the occurrence of the stereotypic barbering behavior, as they constitute the “recipients” of the behavioral endpoint. However, the actual stereotype is present in the “barbers” but not in the barbered mice.

Based upon these results it is conceivable that the possibility to interact with a multitude of olfactory signals from a variety of individual animals constitutes a form of “olfactory enrichment” to substitute for the reduced options mice within the traditional cage environment are given to satisfy their exploratory drive. Indeed, the fact that laboratory animals are provided with all requirements for physical wellbeing (water, food, shelter, etc.), means that in the spare time–which they have plenty of in a laboratory environment–they switch to information gathering–or exploratory behavior (Young, 2008). As such, the odor cues contained in the soiled bedding may, as element of enrichment, serve to enhance the animals’ species-specific behavior, hereby helping to “improve animal welfare and the behavioral and physiological integrity of the animal (e.g., decreasing abnormal behavior and increasing the animal’s ability to cope with the challenges of captivity and experimentation)” (Olsson et al., 2003).

N/L ratio has been used as proxy for a subject’s response to the experience of chronic stress in various animals, including laboratory mice (Davis et al., 2008; Swan and Hickman, 2014; Hickman, 2017). The assumption that the relative deprivation of sensorial stimulation within the conventional laboratory cage environment can be considered as a constant challenge situation in which the animals need to permanently contrast their inherent drives (e.g., foraging, exploration, experience of large social communities, burrowing etc.) with the limited offer of their daily environment (Cait et al., 2022). Against this background we used the N/L ratio as reductionistic approximation to the animal’s internal challenge state, albeit without further concomitant (behavioral, activity) measurements, neither a positive nor a negative valence of this condition can be assumed. However, the finding of decreased within-group variability of the N/L ratio indicates that soiled bedding exposure reduces the individualization of the animals’ responses to the constant challenges of life within the conventional laboratory cage. One interpretation could be that a large part of variability within the group is driven by those animals displaying stereotypic behavior, likely consequent to, or reflecting a low ability to cope with their unmet physiological needs. Hence, more animals with stereotypic behaviors would therefore increase the within-group variability. This hypothesis is supported by our observation that bar-mouthing behavior and barbering are increased in the control, as compared to the soiled bedding group. Interestingly, other enrichment strategies have been found to drive individualization of selected behavioral and physiological traits, an effect interpreted as activity-dependent development of brain individuality (Freund et al., 2013) particularly in experiments with large environments and/or time scales (days). Hence, the reduction in inter-individual variability upon long-term soiled bedding exposure is not a consequence of enhanced activity-induced plasticity. Rather it may reflect a reduction of the inter-individual data spread due to reduced concomitant pathology under the housing condition with soiled bedding exposure.

While more experiments, including various other behavioral (such as general and exploratory activity) and physiological read-outs are needed to corroborate this analysis and define its relevance in different contexts, we propose that by reducing within-group variability, “olfactory enrichment” may increase statistical power, thus reducing the number of female mice needed in this specific experiment.

Further research is warranted to define the optimal composition (litter versus bedding), amount, source (number and sex of animals) of soiled bedding to most beneficially modulate animal welfare. Specifically, it is important to highlight that the present study employed only female mice exposed to used bedding of female mice and future studies will be needed to determine if and these results have to be interpreted in a sex-dependent manner. However, our data firstly suggest that olfactory cues may enrich the cage environment to exert positive effects on the well-being of laboratory animals and, by reducing between-animal variability, increase homogeneity of experimental results, hereby possibly reducing the statistical requirements of the number of experimental animals needed.

## Data availability statement

The raw data supporting the conclusions of this article will be made available by the authors, without undue reservation.

## Ethics statement

The study was approved by the ethics and animal welfare committee of the Medical University of 105 Vienna in accordance with Good Scientific Practice guidelines and national legislation: licence number 106 BMBWF-66.009/0257-V/3b/2018.

## Author contributions

KM performed experiments and analyzed data. TL conceived the project. JK-P performed experiments. JW and RP procured funding. LY and CK analyzed data. SK designed behavioral experiments. RPl procured funding and conceived the project. KT and DDP wrote the manuscript. All authors contributed to the article and approved the submitted version.
