# Supplementary material for: Exposure to soiled bedding reduces abnormal repetitive behaviors in mice
Source: Front Behav Neurosci. 2022 Nov 22;16:1062864. doi: 10.3389/fnbeh.2022.1062864 (PMC9722949; doi:10.3389/fnbeh.2022.1062864)
Supplement: Supplementary file 1 [file Data_Sheet_1.PDF]

# *Supplementary Material*

## 1 Supplementary methods

Collection and analysis of fecal samples.

To access individual samples for FCM, FS cages were custom-developed and built, adapted from a special fecal collection cage for spiny mice (Frynta et al., 2009) (Supplementary Figure 1). These cages allow collecting individual feces samples without touching the animals, to enable simultaneous sampling of all animals within a cage and for all at the same time point. FCM levels vary strongly with the diurnal rhythm (Touma et al., 2004) and sampling for FCM was carried out between 7 and 9 p.m.

under red light conditions.

After collection, the samples were thoroughly homogenized and frozen at -20°Ci n 0.05 g aliquots until analysis. After thawing, 1 ml 80% methanol12 was added and samples were vortexed and centrifuged. The supernatant was diluted 1:20 with assay buffer (Tris/HCl 20 mM, pH 7.5). FCMs were measured with a 5α-pregnane-3β, 11β, 21-triol-20-one enzyme immunoassay (EIA) (Touma et al., 2003).

## 2 Supplementary Figures and Tables

### 2.1 Supplementary Table 1 - Results of all calculated mixed models (Estimate 95% CI, df, p-value).

| Results for mixed models | | | |
|---|---|---|---|
| | **Estimate [95% CI]** | **d.f.** | **p-value** |
| **N:L - Ratio:** FS cage vs. normal cage*soiled bedding vs. control group, random effect cage number | | | |
| (Intercept) | 0.413 [0.250, 0.577] | 35.330 | 0.000 |
| GroupTreatment | -0.103 [-0.331,0.126] | 33.145 | 0.385 |
| CT FS cage | -0.105 [-0.327,0.117] | 33.647 | 0.360 |
| GroupTreatment:CT FS cage | 0.035 [-0.285,0.355] | 32.201 | 0.832 |
| **FCM:** time*soiled bedding vs. control group, mouse nested in cage number | | | |
| (Intercept) | 157.696 [136.012,179.379] | 59.265 | 0.000 |
| time | -8.264 [-15.639,-0.889] | 96.991 | 0.030 |
| GroupTreatment | -7.320 [-39.121,24.480] | 66.100 | 0.653 |
| time:GroupTreatment | 4.619 [-6.134,15.372] | 100.809 | 0.402 |
| **Barbering:** time*soiled bedding vs. control group, random effect cage number | | | |
| (Intercept) | 0.052 [0.057,0.160] | 43.341 | 0.358 |
| time | 0.007 [0.002,0.012] | 358.000 | 0.004 |
| GroupTreatment | -0.046 [-0.200,0.108] | 43.341 | 0.561 |
| time:GroupTreatment | 0.016 [0.009,0.023] | 358.000 | 0.000 |
| **Bar mouthing:** time*soiled bedding vs. control group, mouse nested in cage number | | | |
| (Intercept) | 1.44 [0.895,1.989] | | 0.000 |
| GroupTreatment | -0.918 [-1.734,-0.102] | | 0.027 |
| time | 0.196 [0.112,0.280] | | 0.000 |

| | | | |
|---|---|---|---|
| time:GroupTreatment | 0.128 [-0.016,0.271] | | 0.081 |
| **Circling:** time*soiled bedding vs. control group, mouse nested in cage number | | | |
| (Intercept) | 121.250 [29.681,212.819] | 79.988 | 0.011 |
| GroupTreatment | -73.133 [-202.631,56.365] | 79.988 | 0.272 |
| time | -20.608 [-48.875,7.658] | 70.000 | 0.157 |
| time:GroupTreatment | 34.950 [-5.025,74.925] | 70.000 | 0.091 |
| **Social grooming:** time*soiled bedding vs. control group, mouse nested in cage number | | | |
| (Intercept) | 2.083 [-65.238,69.405] | 36.930 | 0.952 |
| GroupTreatment | -34.900 [-130.106,60.307] | 36.930 | 0.477 |
| time | 10.047 [-11.383,31.476] | 86.000 | 0.361 |
| GroupTreatment:time | 33.020 [2.714,63.326] | 86.000 | 0.036 |
| **Self grooming:** time*soiled bedding vs. control group, mouse nested in cage number | | | |
| (Intercept) | 167.117 [69.095,265.138] | 62.597 | 0.001 |
| GroupTreatment | 47.117 [-91.507,185.740] | 62.597 | 0.508 |
| time | 5.155 [-29.833,40.143] | 70.000 | 0.774 |
| GroupTreatment:time | 13.325 [-36.155,62.805] | 70.000 | 0.599 |
| **Weight:** time*soiled bedding vs. control group, random effect cage number | | | |
| (Intercept) | 19.102 [18.522,19.681] | 110.480 | 0.000 |
| time | 1.589 [1.437,1.741] | 358.000 | 0.000 |
| GroupTreatment | -0.468 [-1.308,0.372] | 110.480 | 0.277 |
| time:GroupTreatment | 0.064 [-0.156,0.285] | 358.000 | 0.567 |
| **Mounting:** time*soiled bedding vs. control group, mouse nested in cage number | | | |
| (Intercept) | 1.263 [-0.103,2.629] | | 0.070 |
| GroupTreatment | -0.928 [-2.805,0.950] | | 0.333 |
| time | -1.054 [-1.446,-0.662] | | 0.000 |
| GroupTreatment:time | 0.530 [0.049,1.012] | | 0.031 |
| **Chasing:** time*soiled bedding vs. control group, mouse nested in cage number | | | |
| (Intercept) | -0.909 [-2.307,0.489] | | 0.202 |
| GroupTreatment | -0.092 [-2.193,2.009] | | 0.931 |
| time | -0.363 [-0.826,0.100] | | 0.124 |
| GroupTreatment:time | -0.177 [-1.014,0.659] | | 0.678 |

## 2.2 Supplementary Figure 1

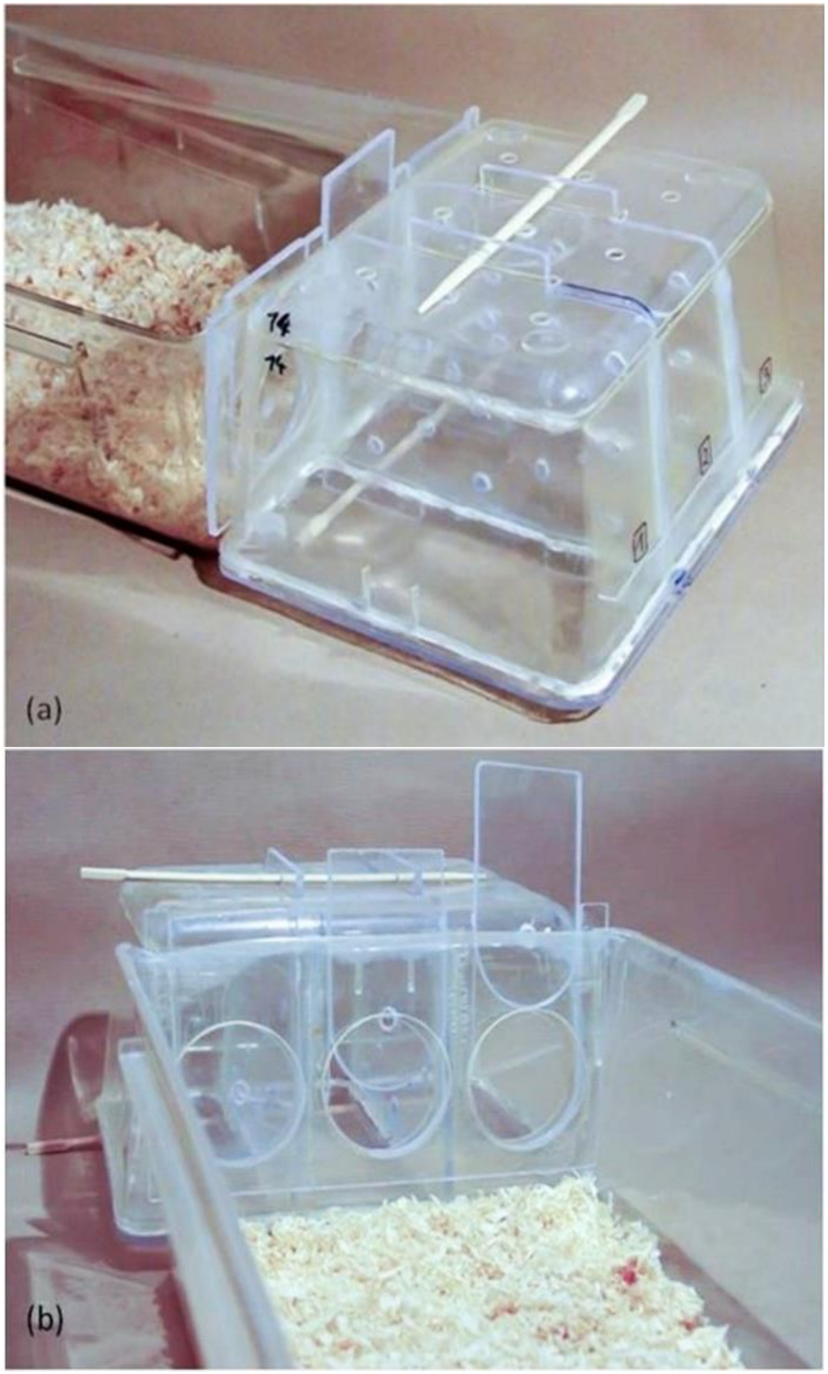


**Supplementary Figure 1. Custom-made cage for fecal sample collection; a)** The collection cage is divided into three boxes for the collection of samples from the individual animals and covered by a lid to be opened for removal of the samples. **b)** View from inside the cage with the doors that were opened at the start of the sampling and closed as soon as the mice were inside the box.
